# Identification of Informed Consent in Patient Videos on Social Media: Prospective Study

**DOI:** 10.2196/14081

**Published:** 2020-10-13

**Authors:** Jane O'Sullivan, Cathleen McCarrick, Paul Tierney, Donal B O'Connor, Jack Collins, Robert Franklin

**Affiliations:** 1 Department of Anaesthesia Letterkenny University Hospital Donegal Ireland; 2 Department of Surgery Tallaght University Hospital Dublin Ireland; 3 Department of Anatomy Trinity College Dublin Dublin Ireland; 4 Professorial Surgical Unit Tallaght University Hospital & Trinity College Dublin Dublin Ireland

**Keywords:** social media, patient consent, patient footage, ethics, YouTube, patient video, medical education

## Abstract

**Background:**

The American Medical Association Code of Medical Ethics states that any clinical image taken for public education forms part of the patient’s records. Hence, a patient’s informed consent is required to collect, share, and distribute their image. Patients must be informed of the intended use of the clinical image and the intended audience as part of the informed consent.

**Objective:**

This paper aimed to determine whether a random selection of instructional videos containing footage of central venous catheter insertion on real patients on YouTube (Google LLC) would mention the presence of informed consent to post the video on social media.

**Methods:**

We performed a prospective evaluation by 2 separate researchers of the first 125 videos on YouTube with the search term “central line insertion.” After duplicates were deleted and exclusion criteria applied, 41 videos of patients undergoing central line insertion were searched for reference to patient consent. In the case of videos of indeterminate consent status, the posters were contacted privately through YouTube to clarify the status of consent to both film and disseminate the video on social media. A period of 2 months was provided to respond to initial contact. Furthermore, YouTube was contacted to clarify company policy. The primary outcome was to determine if videos on YouTube were amended to include details of consent at 2 months postcontact. The secondary outcome was a response to the initial email at 2 months.

**Results:**

The researchers compiled 143 videos. Of 41 videos that contained footage of patient procedures, 41 were of indeterminate consent status and 23 contained identifiable patient footage. From the 41 posters that were contacted, 3 responded to initial contact and none amended the video to document consent status. Response from YouTube is pending.

**Conclusions:**

There are instructional videos for clinicians on social media that contain footage of patients undergoing medical procedures and do not have any verification of informed consent. While this study investigated a small sample of available videos, the problem appears ubiquitous and should be studied more extensively.

## Introduction

The primacy of YouTube (Google LLC) as a learning tool used by health care professionals cannot be overestimated. In a study published in 2016 by Rapp et al [1], a survey was distributed to surgical consultants and trainees, which established that 90% of all respondents reported using videos as a learning resource prior to performing a surgical procedure. Of those that used videos, 86% reported using YouTube as the resource. Medical practitioners have a duty to ensure that the information made available for use on YouTube has been sourced in an ethical fashion.

The American Medical Association (AMA) Code of Medical Ethics states that any clinical image taken for public education forms part of the patient’s record. Hence, a patient’s informed consent is required to collect, share, and distribute their image. Patients must be informed of the intended use of the clinical image and the intended audience as part of the informed consent [2]. Therefore, it is not sufficient to obtain permission to use the videos for educational purposes. A critical component of informed consent must include explaining to the patient that the video will be posted on YouTube.

The purpose of this study is to determine if a random selection of step-by-step instructional, procedural videos involving patients and posted on YouTube indicate the patient’s consent. Furthermore, in the case of an indeterminate consent status, it seeks to clarify whether the poster or trainer obtained informed consent for the production and dissemination online of the video. The overall objective is to determine if there is an issue with patient consent status on these YouTube videos. It serves to provide insight into a potential problem that indeed may be widespread.

## Methods

A common clinical procedure was selected for the purpose of the study: central line insertion. On 2 separate occasions, 2 independent researchers searched the term “central line insertion” on YouTube. Each researcher formulated a list of the first 125 videos for the search term. The lists were then collated, and any video duplicates were deleted.

Each researcher separately analyzed every video included on the list, extracting the necessary details. The included videos were instructional in nature, giving a step-by-step account of how to insert a central line. For the purpose of this study, the following exclusion criteria were applied: non–English-speaking videos, simulation procedures, animated procedures, blogs, animal procedures, and any videos that did not show the actual procedures being performed. All English language videos of patients undergoing central line insertion were included.

For those videos meeting the inclusion criteria, each was searched for any reference to patient consent. They were additionally analyzed to determine if the patient was identifiable. Patients were deemed identifiable if the face anterior to the tragus of the external ear was visible. Finally, the video was evaluated for any details pertaining to that patient’s care.

In the case of videos of indeterminate consent status, the posters were contacted privately through YouTube. Furthermore, the videos were analyzed for any contact details for the trainer. An email was sent to them, including a brief introduction and an inquiry as to whether consent was obtained to film this video and post it on social media. A period of 15 days was allowed to elapse before checking the videos again to determine if they had been updated to include information regarding the patient’s consent. The primary outcome of the study was to determine whether the YouTube posters included details about patients’ consent to post the video on social media. The secondary outcome was whether the poster responded to the private message and amended their videos to clarify the consent status of the patient. The videos were re-examined after a further period of 2 months to determine if they had been updated to include information regarding the consent status of the patient displayed in the video.

The following email was sent to each of the posters:

Hi, We are a group of researchers from Ireland. We are completing a project on consent for YouTube videos involving patients. We hoped that we could ask you several brief questions. Did you receive patient consent prior to the production and distribution of this video? If so, what was the form of the consent? Are you aware of any guidelines that govern the consent process for posting patient videos on YouTube? If you have consent, would you consider mentioning the consent on the video following this email? Thank you very much for taking the time to read this.

Finally, contact was made with YouTube regarding its policy surrounding patient consent. The email to YouTube highlighted the list of patient videos of indeterminate consent status. It noted the AMA guidelines and requested that YouTube clarify the matter. The following email was sent to YouTube:

Dear YouTube, My colleagues and I are medical doctors in Ireland. We are currently undertaking a research project on patient consent on social media. We noticed that a number of videos posted contain footage of real patients undergoing medical procedures in healthcare facilities. We have examples of videos which contain identifiable and non-identifiable imagery of the patients. The British General Medical Council state that any person posting videos containing real patient procedures must seek prior written consent, regardless of whether the patient is identifiable. We have made a large database of videos that contravene these guidelines. We contacted the posters of these videos and gave them a two-week period to respond. There was minimal response to our queries. We would be obliged if you could clarify your stance on allowing videos with indeterminate consent to be posted on YouTube, in terms of your current policy.

## Results

**Figure 1:**
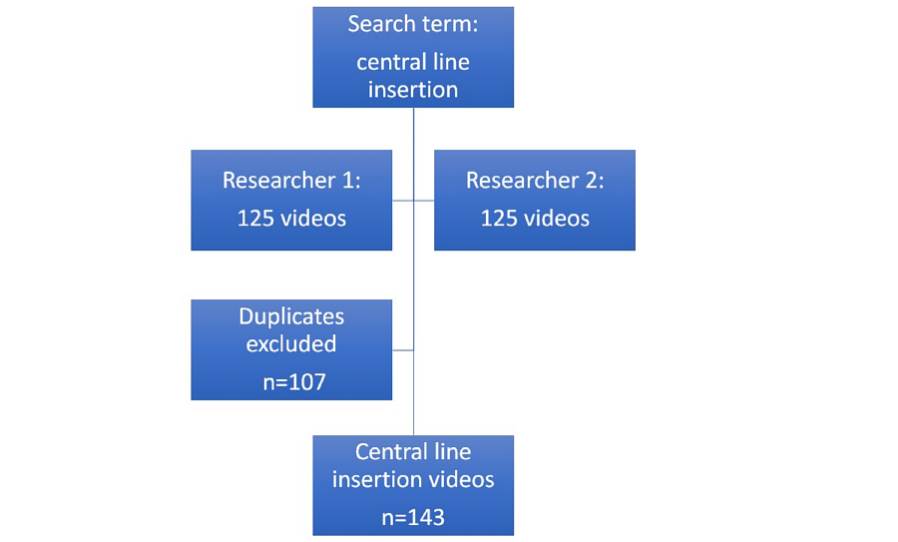
Search results on YouTube for the search term "central line insertion." Each researcher performed a separate search on different days using the defined search term.

The search term “central line insertion” was input into the YouTube search engine. Both researchers separately identified the first 125 videos for this search term. When the researchers’ lists were combined, there were 104 duplicate entries. After duplicates were removed and the researchers’ results were combined, there were 143 videos of central line insertion in total. This process can be seen in Figure 1.

**Figure 2:**
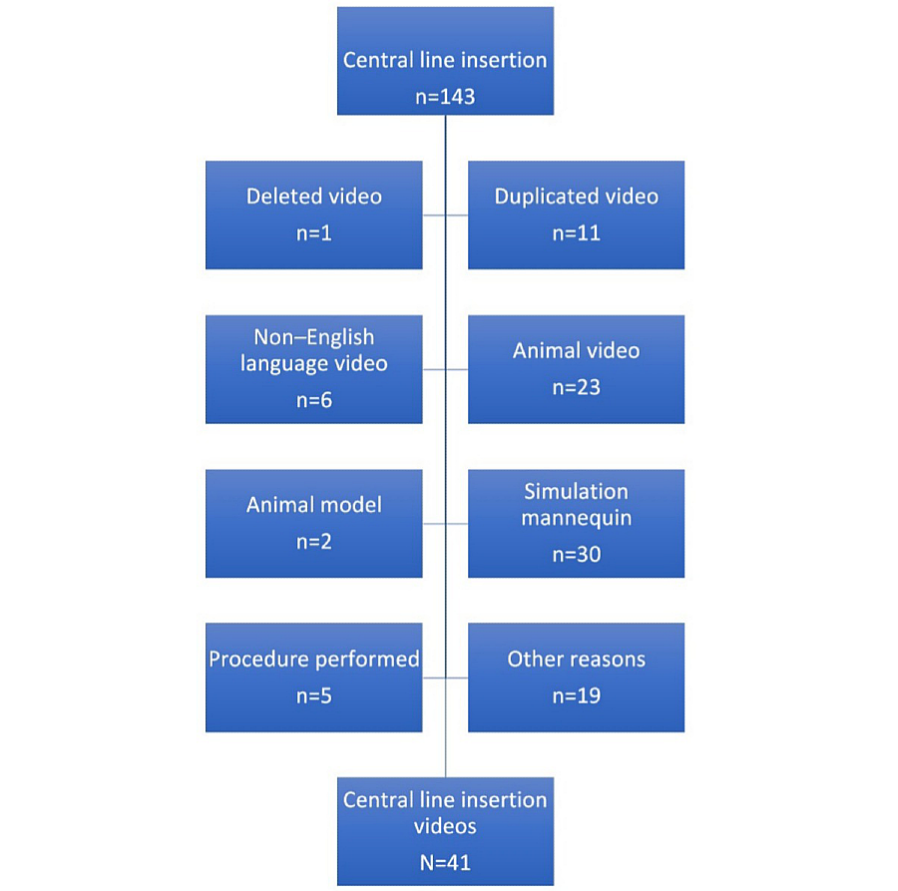
Final compilation of central line insertion videos after exclusion of videos based on the exclusion criteria.

The remaining YouTube videos were scrutinized to determine if they fulfilled the inclusion criteria, as seen in Figure 2. A total of 102 of these videos failed to meet the inclusion criteria for various reasons, as discussed in the “Methods” section. After excluding these videos, the researchers were left with 41 videos in total of clinician-led video entries detailing a central line insertion on a real patient.

Each of the 41 clinicians who posted a video on YouTube was contacted via the private message function on YouTube. Only 3 of the 41 posters responded to the email. Following reanalysis of the videos 2 months postcontact, 0 of the 41 posters amended the original video to state whether there was any patient consent obtained prior to posting the video on YouTube. Additionally, 0 of the 41 videos mentioned the original trainer.

All 3 posters who responded were clinicians. One of the respondents stated that written consent was obtained to use the video for educational purposes. This respondent failed to state whether informed consent was obtained for uploading the video onto social media. Another poster stated that the particular institution he worked at did not mandate informed consent for the production and posting of videos on social media as long as there were no patient identifiers. The third poster stated that verbal consent was obtained to post the video on YouTube.

Of these 41 videos, the anterior face was visible in 56% (23/41) of the YouTube videos. Anterior face was defined as any part of the face anterior to the tragus. Anterior face was taken as a surrogate marker for identifiable patients.

YouTube has yet to respond to the email aiming to clarify the company’s policy on the posting of patient-containing footage.

## Discussion

This study examined a random selection of videos (n=41) of a common clinical procedure that contained real patient footage of indeterminate consent status. Of these, 56% (23/41) showed potentially identifiable patient footage. Only 3 posters responded to the email designed to clarify the consent status of the published video. All 3 posters were physicians.

For the purpose of this study, any image showing the face anterior to the tragus of the external ear was deemed identifiable. Stieber et al [3] specified an identifiable patient image as any patient image that contains sufficient information to enable a non–medically trained individual to correctly identify the patient or that is readily identifiable to the patient themself. Although each institution may have its own specified standards as to what constitutes an identifiable image, a nonidentifiable image must not meet either of the above criteria, which casts doubt on the legal validity of individual institutional standards.

Informed consent may be defined as “autonomous authorization by a patient or subject” [4]. There are different levels of patient consent. While a patient may agree to allow an image to be recorded for the purpose of their medical notes, they may not necessarily agree for this image to be disseminated on social media [5]. The concept of consent must continue to evolve to encapsulate all the challenges posed by modern technology. The videos included in the study contained reference to neither the patient’s consent to undergo the procedure nor to their consent to the publication of these videos on social media platforms.

Social media is defined as a website or application that allows users to generate or upload content or to engage in social networking. The differentiating factor between social media and a website is the ability of a user to use and redistribute the uploaded material freely on social media. Generally, content on a website is restricted due to copyright considerations. Furthermore, content uploaded onto a social media platform is usually shared instantaneously with viewers or followers. Finally, social media engenders interactive participation and discussion of the material [6]. As a consequence, patient information posted to social media spreads a lot more rapidly and widely than content on a website.

There are several pitfalls associated with the use of social media in health care. The posting clinician forfeits sole control and ownership of the material posted on social media and the ability to delete material once posted. Such issues need to be discussed with the patient prior to obtaining informed consent. Furthermore, in normal circumstances, informed consent is a dynamic process. Consequently, the patient has the right to withdraw this informed consent at any stage in the process. However, in the case of social media it is virtually impossible to remove images and hence, informed consent is invalidated [7]. There are no regulatory mechanisms to ensure that the images are not widely viewed, disseminated, or misused [5]. In order to meet the definition of informed consent, the patient should be made aware of such risks. It is not enough to obtain consent from them to record a video. The patient needs to be made aware of potential consequences relating to the publication of a video on social media. Not only does this paper fail to clarify if patients consented to the recording of procedural footage, it fails to determine in all videos posted if patients were informed of potentially negative outcomes of broadcasting a video on social media.

Physicians are under obligation to inform patients about any procedure being contemplated. In the legal domain, when informed consent is breached, the breach must satisfy the following 4 criteria in order to be deemed negligent: (1) the physician must fail to disclose this information about the procedure to the patient, (2) there must be consequences for the patient that causes the patient to be worse off, (3) the adverse outcome is a consequence of the physician’s failure to disclose the information to the patient regarding the procedure, and (4) had the patient been aware of the risk, they would not have consented to the procedure [4]. In the case of videos containing identifiable patient material in the absence of consent to publish on social media, all 4 of the above criteria are satisfied if harm is defined in terms of psychological damage. Thus, it would be possible to argue malpractice in instances of foregoing consent where identifying features are present.

The World Medical Association Declaration of Lisbon on the Rights of the Patient states that irrespective of geographical location, all patients have the right to information and self-determination [8]. Despite this guideline, there is considerable cultural variation in both the practice of informed consent and the salience of informed consent with respect to patient autonomy. Cultural differences, however, should not abrogate the need for informed consent [9]. Irrespective of patient location, basic ethical benchmarks should apply to patients of all jurisdictions and circumstances. Furthermore, this footage is being used by practicing clinicians in jurisdictions where there are ethical concerns regarding the filming of patients. These clinicians should ensure that their educational materials are ethically sourced.

The source of the video material is not always identifiable. In a recent study by Pitcher and Amendolo [10] that analyzed videos of common femoral artery access published on YouTube, 40% (13/33) of videos were published by unknown practitioners. For the majority of the videos included in this study, it was not possible to determine the source of the information, which emphasizes the poster’s loss of control of material uploaded onto social media platforms.

Bezner and colleagues [11] examined the first 40 English language videos of 4 different pediatric diagnoses published on YouTube. The researchers noted that a limiting factor to the use of YouTube for accessing patient videos was the absence of information surrounding the patient’s consent to film and distribute the video on social media. None of the videos included in their study specified this consent. Similarly, in this study, prior to contacting the posters, no video referred to the patient’s informed consent to film the procedure. Following contact with the posters, the consent status was available for only 3 videos. The remainder of the videos were indeterminate as to consent status.

Following the results of this study, it is clear that contacting those who have posted videos on YouTube is an ineffective way of obtaining the consent status of the video. The emails sent to the users yielded poor results. The vast majority (38/41) of the central line insertion videos remain of unknown consent status. Thus, videos that are viewed every day by medical practitioners may not meet sufficiently rigorous ethical criteria. It would seem necessary that those posting videos on YouTube need to ensure that their patients have given informed consent, as in the case of medical journals, and that this consent is specified in the uploaded material. Such solutions would require governance by an external body, however. It may be necessary to establish a clinical governance group to monitor social media content in collaboration with YouTube. Following the submission of this paper for publication, the authors are still awaiting a response on YouTube’s policy.

A limitation of the present study is that a small selection of videos (n=143) was examined for a single procedure. One cautions against extrapolating the present results to other fields. However, the purpose of this study was to highlight a potential ethical issue of posting videos of patients undergoing procedures on social media. Further work is needed to elucidate whether this is problematic on a wider scale and how this problem can be overcome.

In conclusion, the present study serves to highlight the indeterminate consent status of randomly selected, patient-containing footage on YouTube.

## Abbreviations

AMA: American Medical Association.
