# Correction: Identification of Informed Consent in Patient Videos on Social Media: Prospective Study

**DOI:** 10.2196/25045

**Published:** 2020-10-30

**Authors:** Jane O'Sullivan, Cathleen McCarrick, Paul Tierney, Donal B O'Connor, Jack Collins, Robert Franklin

**Affiliations:** 1 Department of Anaesthesia Letterkenny University Hospital Donegal Ireland; 2 Department of Surgery Tallaght University Hospital Dublin Ireland; 3 Department of Anatomy Trinity College Dublin Dublin Ireland; 4 Professorial Surgical Unit Tallaght University Hospital & Trinity College Dublin Dublin Ireland

In “Identification of Informed Consent in Patient Videos on Social Media: Prospective Study” (JMIR Med Educ 2020;6(2):e14081) the authors noted one error.

The corresponding author affiliation was inadvertently published with the incorrect phone number. The correct phone number has now been added as follows: 353 1 414 2000.

The correction will appear in the online version of the paper on the JMIR Publications website on October 30, 2020, together with the publication of this correction notice. Because this was made after submission to PubMed, PubMed Central, and other full-text repositories, the corrected article has also been resubmitted to those repositories.

